# Supplementary material for: Top-down feedback in deep neural networks leads to functional differences during audiovisual integration
Source: eLife. 2026 Aug 26;14:RP105953. doi: 10.7554/eLife.105953 (PMC13516690; doi:10.7554/eLife.105953)
Supplement: Peer review history

# Editor report: eLife Assessment

**DOI:** 10.7554/eLife.105953.3.sa0

**Authors:** Julijana Gjorgjieva

**Affiliations:** Technical University of Munich Germany

**Keywords:** Solid, Valuable

This **valuable** study investigates the computational role of top-down feedback - a property found in biological circuits - in artificial neural network (ANN) models of the neocortex. Using hierarchical recurrent ANNs in an audiovisual integration task, the authors show that an anatomically inspired feedback motif induces a stable visual bias consistent with human perception and yields modest but meaningful benefits for learning dynamics and robustness. The strength of evidence is **solid**: the modeling, analyses, and controls mostly support the central claim that top-down feedback motifs impose persistent inductive biases that shape functional specialization and behavior. But the evidence for a broad, general framework that predicts behavior remains only partially supported, and the Methods would benefit from a compact, reproducible summary of hyperparameters and architectural details.

# Referee report: Reviewer #1 (Public review)

**DOI:** 10.7554/eLife.105953.3.sa1

Summary:

Here, the authors aim to investigate the potential improvements of ANNs when used to explain brain data using top-down feedback connections found in the neocortex. To do so, they use a retinotopic and tonotopic organization to model each subregion of the ventral visual (V1, V2, V4, and IT) and ventral auditory (A1, Belt, A4) regions using Convolutional Gated Recurrent Units. The top-down feedback connections are inspired by the apical tree of pyramidal neurons, modeled either with a multiplicative effect (change of gain of the activation function) or a composite effect (change of gain and threshold of the activation function).

To assess the functional impact of the top-down connections, the authors compare three architectures: a brain-like architecture derived directly from brain data analysis, a reversed architecture where all feedforward connections become feedback connections and vice versa, and a random connectivity architecture. More specifically, in the brain-like model the visual regions provide feedforward input to all auditory areas, whereas auditory areas provide feedback to visual regions.

First, the authors found that top-down feedback influences audiovisual processing and that the brain-like model exhibits a visual bias in multimodal visual and auditory tasks. Second, they discovered that in the brain-like model, the composite integration of top-down feedback, similar to that found in the neocortex, leads to an inductive bias toward visual stimuli, which is not observed in the feedforward-only model. Furthermore, the authors found that the brain-like model learns to utilize relevant stimuli more quickly while ignoring distractors. Finally, by analyzing the activations of all hidden layers (brain regions), they found that the feedforward and feedback connectivity of a region could determine its functional specializations during the given tasks.

Strengths:

The study introduces a novel methodology for designing connectivity between regions in deep learning models. The authors also employ several tasks based on audiovisual stimuli to support their conclusions. Additionally, the model utilizes backpropagation of error as a learning algorithm, making it applicable across a range of tasks, from various supervised learning scenarios to reinforcement learning agents. Conversely, the presented framework offers a valuable tool for studying top-down feedback connections in cortical models. Thus, it is a very nice study that can also give inspiration to other fields (machine learning) to start exploring new architectures.

# Referee report: Reviewer #2 (Public review)

**DOI:** 10.7554/eLife.105953.3.sa2

Summary:

This work addresses the question whether artificial deep neural network models of the brain could be improved by incorporating top-down feedback, inspired by the architecture of neocortex.

In line with known biological features of cortical top-down feedback, the authors model such feedback connections with both, a typical driving effect and a purely modulatory effect on the activation of units in the network.

To asses the functional impact of these top-down connections, they compare different architectures of feedforward and feedback connections in a model that mimics the ventral visual and auditory pathways in cortex on an audiovisual integration task.

Notably, one architecture is inspired by human anatomical data, where higher visual and auditory layers possess modulatory top-down connections to all lower-level layers of the same modality, and visual areas provide feedforward input to auditory layers, whereas auditory areas provide modulatory feedback to visual areas.

First, the authors find that this brain-like architecture imparts the models with a light visual bias similar to what is seen in human data, which is the opposite in a reversed architecture, where auditory areas provide feedforward drive to the visual areas.

Second, they find that, in their model, modulatory feedback should be complemented by a driving component to enable effective audiovisual integration, similar to what is observed in neural data.

Overall, the study shows some possible functional implications when adding feedback connections in a deep artificial neural network that mimic some functional aspects of visual perception in humans.

Strengths:

The study contains innovative ideas, such as incorporating an anatomically inspired architecture into a deep ANN, and comparing its impact on a relevant task to alternative architectures.

Moreover, the simplicity of the model allows it to draw conclusions on how features of the architecture and functional aspects of the top-down feedback affects performance of the network.

This could be a helpful resource for future studies of the impact of top-down connections in deep artificial neural network models of neocortex.

Weaknesses:

Some claims not yet supported.

The problem is that results are phrased quite generally in the abstract and discussion, while the actual results shown in the paper are very specific to certain implementations of top-down feedback and architectures. This could lead to misunderstanding and requires some revisions of the claims in the abstract and discussion (see below).

"Altogether our findings demonstrate that modulatory top-down feedback is a computationally relevant feature of biological brain..."

This claim is not supported, since no performance increase is demonstrated for modulatory feedback. So far, only the second half of the sentence is supported: "...and that incorporating it into ANNs affects their behavior and constrains the solutions it's likely to discover."

"This bias does not impair performance on the audiovisual tasks."

This is only true for the composite top-down feedback that combines driving and modulatory effects, whereas modulatory feedback alone can impair the performance (e.g., in the visual tasks VS1 and VS2). The fact that modulatory feedback alone is insufficient in ANNs to enable effective cross-modal integration and requires some driving component is actually very interesting, but it is not stressed enough in the abstract. This is hinted at in the following sentence, but should be made more explicitly:

"The results further suggest that different configurations of top-down feedback make otherwise identically connected models functionally distinct from each other, and from traditional feedforward and laterally recurrent models."

"Here we develop a deep neural network model that captures the core functional properties of top-down feedback in the neocortex" -> this is too strong, take out "the", because very likely there are other important properties that are not yet incorporated.

"Altogether, our results demonstrate that the distinction between feedforward and feedback inputs has clear computational implications, and that ANN models of the brain should therefore consider top-down feedback as an important biological feature."

This claim is still not substantiated by evidence provided in the paper. First, the wording is a bit imprecise, because mechanistically, it is not really the feedforward versus feedback (a purely feedforward model is not considered at all in the paper), but modulatory versus driving. Moreover, the second part of the sentence is problematic: The results imply that, computationally/functionally, driving connections are doing the job, while modulatory feedback does not really seem to improve performance (best case, it does not do any harm). It is true that it is a feature that is inspired by biology, but I don't see why the results imply that (modulatory) top-down feedback should be considered in ANN models of the brain. This would require to show that such models either improve performance, or do improve the ability to fit neural data, both which are beyond the scope of the paper.

The same argument holds for the following sentence, which is not supported by the results of the paper:

"More broadly, our work supports the conclusion that both the cellular neurophysiology and structure of feed-back inputs have critical functional implications that need to be considered by computational models of brain function."

Additional supplementary material required

Although the second version checked the influence of processing time, this was not done for the most important figure of the paper, Figure 4. A central claim in the abstract "This bias does not impair performance on the audiovisual tasks" relies on this figure, because only with composite feedback the performance is comparable between the the "drive-only" and "brain-like" models. Thus, the supplementary Figure 3 should also include the composite networks and drive only network to check the robustness of the claim with respect to process time. This robustness analysis should then also be mentioned in the text. For example, it should be mentioned whether results in these networks are robust or not with respect to process time, whether there are differences between network architectures or types of feedback in general etc.

Moreover, the current analysis for networks with modulatory feedback is a bit confusing. Why is the performance so low for the reverse model for a process time of 3 and 10? This is a very strong effect that warrants explanation. More details should be added in the caption as well. For example, are the models separately trained for the output after 3 and 10 processing steps for the comparison, or just evaluated at these times? Not training these networks separately might explain the low performance for some networks, so ideally networks are trained for each choice of processing steps.

# Referee report: Reviewer #3 (Public review)

**DOI:** 10.7554/eLife.105953.3.sa3

Summary:

This study investigates the computational role of top-down feedback in artificial neural networks (ANNs), a feature that is prevalent in the brain but largely absent in standard ANN architectures. The authors construct hierarchical recurrent ANN models that incorporate key properties of top-down feedback in the neocortex. Using these models in an audiovisual integration task, they find that hierarchical structures introduce a mild visual bias, akin to that observed in human perception, not always compromising task performance.

Strengths:

The study investigates a relevant and current topic of considering top-down feedback in deep neural networks. In designing their brain-like model, they use neurophysiological data, such as externopyramidisation and hierarchical connectivity. Their brain-like model exhibits a visual bias that qualitatively matches human perception.

Weaknesses:

While the model is brain-inspired, it has limited bioplausibility. The model assumes a simplified and fixed hierarchy. The authors acknowledge this limitation in the discussion.

While the brain-like model showed an advantage in ignoring distracting auditory inputs, it struggled when visual information had to be ignored. This suggests that its rigid bias toward visual processing could make it less adaptive in tasks requiring flexible multimodal integration. It hence does not necessarily constitute an improvement over existing ANNs. The study does not evaluate whether the top-down feedback architecture scales well to more complex problems or larger datasets. A valuable future contribution would be to evaluate how the network's behaviour fits to human data.

# Author comment: Author response

**DOI:** 10.7554/eLife.105953.3.sa4

**Authors:** Mashbayar Tugsbayar, Mingze Li, Eilif B Muller, Blake Richards

**Affiliations:** McGill University Montreal Canada; McGill University Montreal Canada; Université de Montréal Montreal Canada; McGill University Montreal Canada

The following is the authors’ response to the original reviews.

**Public Reviews:**

**Reviewer #1 (Public review):**

Summary:

Here, the authors aim to investigate the potential improvements of ANNs when used to explain brain data using top-down feedback connections found in the neocortex. To do so, they use a retinotopic and tonotopic organization to model each subregion of the ventral visual (V1, V2, V4, and IT) and ventral auditory (A1, Belt, A4) regions using Convolutional Gated Recurrent Units. The top-down feedback connections are inspired by the apical tree of pyramidal neurons, modeled either with a multiplicative effect (change of gain of the activation function) or a composite effect (change of gain and threshold of the activation function).

To assess the functional impact of the top-down connections, the authors compare three architectures: a brain-like architecture derived directly from brain data analysis, a reversed architecture where all feedforward connections become feedback connections and vice versa, and a random connectivity architecture. More specifically, in the brain-like model the visual regions provide feedforward input to all auditory areas, whereas auditory areas provide feedback to visual regions.

First, the authors found that top-down feedback influences audiovisual processing and that the brain-like model exhibits a visual bias in multimodal visual and auditory tasks. Second, they discovered that in the brain-like model, the composite integration of top-down feedback, similar to that found in the neocortex, leads to an inductive bias toward visual stimuli, which is not observed in the feedforward-only model. Furthermore, the authors found that the brain-like model learns to utilize relevant stimuli more quickly while ignoring distractors. Finally, by analyzing the activations of all hidden layers (brain regions), they found that the feedforward and feedback connectivity of a region could determine its functional specializations during the given tasks.

Strengths:

The study introduces a novel methodology for designing connectivity between regions in deep learning models. The authors also employ several tasks based on audiovisual stimuli to support their conclusions. Additionally, the model utilizes backpropagation of error as a learning algorithm, making it applicable across a range of tasks, from various supervised learning scenarios to reinforcement learning agents. Conversely, the presented framework offers a valuable tool for studying top-down feedback connections in cortical models. Thus, it is a very nice study that also can give inspiration to other fields (machine learning) to start exploring new architectures.

We thank the reviewer for their accurate summary of our work and their kind assessment of its strengths.

Weaknesses:

Although the study explores some novel ideas on how to study the feedback connections of the neocortex, the data presented here are not complete in order to propose a concrete theory of the role of top-down feedback inputs in such models of the brain.

(1) The gap in the literature that the paper tries to fill in the ability of DL algorithms to predict behavior: "However, there are still significant gaps in most deep neural networks' ability to predict behavior, particularly when presented with ambiguous, challenging stimuli." and "[...] to accurately model the brain."

It is unclear to me how the presented work addresses this gap, as the only facts provided are derived from a simple categorization task that could also be solved by the feedforward-only model (see Figures 4 and 5). In my opinion, this statement is somewhat far-fetched, and there is insufficient data throughout the manuscript to support this claim.

We can see now that the way the introduction was initially written led to some confusion about our goal in this study. Our goal here was not to demonstrate that top-down feedback can enable superior matches to human behaviour. Rather, our goal was to determine if top-down feedback had any real implications for processing ambiguous stimuli. The sentence that the reviewer has highlighted was intended as an explanation for why top-down feedback, and its impact on ambiguous stimuli, might be something one would want to examine for deep neural networks. But, here, we simply wanted to (1) provide an overview of the code base we have created, (2) demonstrate that top-down feedback does impact the processing of ambiguous stimuli.

We agree with the reviewer that if our goal was to improve our ability to predict behaviour, then there was a big gap in the evidence we provided here. But, this was not our goal, and we believe that the data we provide here does convincingly show that top-down feedback has an impact on processing of ambiguous stimuli. We have updated the text in the introduction to make our goals more clear for the reader and avoid this misunderstanding of what we were trying to accomplish here. Specifically, the end of the introduction is changed to:

“To study the effect of top-down feedback on such tasks, we built a freely available code base for creating deep neural networks with an algorithmic approximation of top-down feedback. Specifically, top-down feedback was designed to modulate ongoing activity in recurrent, convolutional neural networks. We explored different architectural configurations of connectivity, including a configuration based on the human brain, where all visual areas send feedforward inputs to, and receive top-down feedback from, the auditory areas. The human brain-based model performed well on all audiovisual tasks, but displayed a unique and persistent visual bias compared to models with only driving connectivity and models with different hierarchies. This qualitatively matches the reported visual bias of humans engaged in audio-visual tasks. Our results confirm that distinct configurations of feedforward/feedback connectivity have an important functional impact on a model's behavior. Therefore, top-down feedback captures behaviors and perceptual preferences that do not manifest reliably in feedforward-only networks. Further experiments are needed to clarify whether top-down feedback helps an ANN fit better to neural data, but the results show that top-down feedback affects the processing of stimuli and is thus a relevant feature that should be considered for deep ANN models in computational neuroscience more broadly.”

(2) It is not clear what the advantages are between the brain-like model and a feedforward-only model in terms of performance in solving the task. Given Figures 4 and 5, it is evident that the feedforward-only model reaches almost the same performance as the brain-like model (when the latter uses the modulatory feedback with the composite function) on almost all tasks tested. The speed of learning is nearly the same: for some tested tasks the brain-like model learns faster, while for others it learns slower. Thus, it is hard to attribute a functional implication to the feedback connections given the presented figures and therefore the strong claims in the Discussion should be rephrased or toned down.

Again, we believe that there has been a misunderstanding regarding the goals of this study, as we are not trying to claim here that there are performance advantages conferred by top-down feedback in this case. Indeed, we share the reviewer’s assessment that the feedforward only model seems to be capable of solving this task well. To reiterate: our goal here was to demonstrate that top-down feedback alters the computations in the network and, thus, has distinct effects on behaviour that need to be considered by researchers who use deep networks to model the brain. But we make no claims of “superiority” of the brain-like model.

In-line with this, we’re not completely sure which claims in the discussion the reviewer is referring to. We note that we were quite careful in our claims. For example, in the first section of the discussion we say:

“Altogether, our results demonstrate that the distinction between feedforward and feedback inputs has clear computational implications, and that ANN models of the brain should therefore consider top-down feedback as an important biological feature.”

And later on:

“In summary, our study shows that modulatory top-down feedback and the architectural diversity enabled by it can have important functional implications for computational models of the brain. We believe that future work examining brain function with deep neural networks should therefore consider incorporating top-down modulatory feedback into model architectures when appropriate.”

If we have missed a claim in the discussion that implies superiority of the brain-like model in terms of task performance we would be happy to change it.

(3) The Methods section lacks sufficient detail. There is no explanation provided for the choice of hyperparameters nor for the structure of the networks (number of trainable parameters, number of nodes per layer, etc). Clarifying the rationale behind these decisions would enhance understanding. Moreover, since the authors draw conclusions based on the performance of the networks on specific tasks, it is unclear whether the comparisons are fair, particularly concerning the number of trainable parameters. Furthermore, it is not clear if the visual bias observed in the brain-like model is an emerging property of the network or has been created because of the asymmetries in the visual vs. auditory pathway (size of the layer, number of layers, etc).

We thank the reviewer for raising this issue, and want to provide some clarifications: First, the number of trainable parameters are roughly equal, since we were only switching the direction of connectivity (top-down versus bottom-up), not the number of connections. We confirmed the biggest difference in size is between models with composite and multiplicative feedback; models with composite feedback have roughly ~1K more parameters, and all models are within the 280K parameter range. We now state this in the methods.

Second, because superior performance was not the goal of this study, as stated above, we conducted limited hyperparameter tuning. Given the reviewer’s comment, we wondered whether this may have impacted our results. Therefore, we explored different hyperparameters for the model during the multimodal auditory tasks, which show the clearest example of the visual dominance in the brainlike model (Figure 3).

We explored different hidden state sizes, learning rates and processing times, and examined whether the core results were different. We found that extremely high learning rates (0.1) destabilize all models and that some models perform poorly under different processing times. But overall, the core results are evident across all hyperparameters where the models learn i.e the different behaviors of models with different connectivities and the visual dominance observed in the brainlike model. We now provide these results in a supplementary figure (Fig. S2, showing larger models trained with different learning rates, and Fig S3, which shows the effect of processing time on AS task performance).

**Reviewer #2 (Public review):**

Summary:

This work addresses the question of whether artificial deep neural network models of the brain could be improved by incorporating top-down feedback, inspired by the architecture of the neocortex.

In line with known biological features of cortical top-down feedback, the authors model such feedback connections with both, a typical driving effect and a purely modulatory effect on the activation of units in the network.

To assess the functional impact of these top-down connections, they compare different architectures of feedforward and feedback connections in a model that mimics the ventral visual and auditory pathways in the cortex on an audiovisual integration task.

Notably, one architecture is inspired by human anatomical data, where higher visual and auditory layers possess modulatory top-down connections to all lower-level layers of the same modality, and visual areas provide feedforward input to auditory layers, whereas auditory areas provide modulatory feedback to visual areas.

First, the authors find that this brain-like architecture imparts the models with a light visual bias similar to what is seen in human data, which is the opposite in a reversed architecture, where auditory areas provide a feedforward drive to the visual areas.

Second, they find that, in their model, modulatory feedback should be complemented by a driving component to enable effective audiovisual integration, similar to what is observed in neural data.

Last, they find that the brain-like architecture with modulatory feedback learns a bit faster in some audiovisual switching tasks compared to a feedforward-only model.

Overall, the study shows some possible functional implications when adding feedback connections in a deep artificial neural network that mimics some functional aspects of visual perception in humans.

Strengths:

The study contains innovative ideas, such as incorporating an anatomically inspired architecture into a deep ANN, and comparing its impact on a relevant task to alternative architectures.

Moreover, the simplicity of the model allows it to draw conclusions on how features of the architecture and functional aspects of the top-down feedback affect the performance of the network.

This could be a helpful resource for future studies of the impact of top-down connections in deep artificial neural network models of the neocortex.

We thank the reviewer for their summary and their recognition of the innovative components and helpful resources therein.

Weaknesses:

Overall, the study appears to be a bit premature, as several parts need to be worked out more to support the claims of the paper and to increase its impact.

First, the functional implication of modulatory feedback is not really clear. The "only feedforward" model (is a drive-only model meant?) attains the same performance as the composite model (with modulatory feedback) on virtually all tasks tested, it just takes a bit longer to learn for some tasks, but then is also faster at others. It even reproduces the visual bias on the audiovisual switching task. Therefore, the claims "Altogether, our results demonstrate that the distinction between feedforward and feedback inputs has clear computational implications, and that ANN models of the brain should therefore consider top-down feedback as an important biological feature." and "More broadly, our work supports the conclusion that both the cellular neurophysiology and structure of feed-back inputs have critical functional implications that need to be considered by computational models of brain function" are not sufficiently supported by the results of the study. Moreover, the latter points would require showing that this model describes neural data better, e.g., by comparing representations in the model with and without top-down feedback to recorded neural activity.

To emphasize again our specific claims, we believe that our data shows that top-down feedback has functional implications for deep neural network behaviour, not increased performance or neural alignment. Indeed, our results demonstrate that top-down feedback alters the behaviour of the networks, as shown by the differences in responses to various combinations of ambiguous stimuli. We agree with the reviewer that if our goal was to claim either superior performance on these tasks, or better fit to neural data, we would need to actually provide data supporting that claim.

Given the comments from the reviewer, we have tried to provide more clarity in the introduction and discussion regarding our claims. In particular, we now highlight that we are not trying to demonstrate that the models with top-down feedback exhibit superior performance or better fit to neural data.

As one final note, yes, the reviewer understood correctly that the “only feedforward” model is a model with only driving inputs. We have renamed the feedforward-only models to drive only models and added additional emphasis in the text to ensure that the distinction is clear for all readers.

Second, the analyses are not supported by supplementary material, hence it is difficult to evaluate parts of the claims. For example, it would be helpful to investigate the impact of the process time after which the output is taken for evaluation of the model. This is especially important because in recurrent and feedback models the convergence should be checked, and if the network does not converge, then it should be discussed why at which point in time the network is evaluated.

This is an excellent point, and we thank the reviewer for raising it. We allowed the network to process the stimuli for seven time-steps, which was enough for information from any one region to be transmitted to any other. We found in some initial investigations that if we shortened the processing time some seeds would fail to solve the task. But, based on the reviewer’s comment, we have now also run additional tests with longer processing times for the auditory tasks where we see the clearest visual bias (Figure 3). We find that different process times do not change the behavioral biases observed in our models, but may introduce difficulties ignoring visual stimuli for some models. Thus, while process time is an important hyperparameter for optimal performance of the model, the central claim of the paper remains. We include this new data in a supplementary figure S3.

Third, the descriptions of the models in the methods are hard to understand, i.e., parameters are not described and equations are explained by referring to multiple other studies. Since the implications of the results heavily rely on the model, a more detailed description of the model seems necessary.

We agree with the reviewer that the methods could have been more thorough. Therefore, we have greatly expanded the methods section. We hope the model details are now more clear.

Lastly, the discussion and testable predictions are not very well worked out and need more details. For example, the point "This represents another testable prediction flowing from our study, which could be studied in humans by examining the optical flow (Pines et al., 2023) between auditory and visual regions during an audiovisual task" needs to be made more precise to be useful as a prediction. What did the model predict in terms of "optic flow", how can modulatory from simple driving effect be distinguished, etc.

We see that the original wording of this prediction was ambiguous, thank you for pointing this out. In the study highlighted (Pines et al., 2023) the authors use an analysis technique for measuring information flow between brain regions, which is related to analysis of optical flow in images, but applied to fMRI scans. This is confusing given the current study, though. Therefore, we have changed this sentence to make clear that we are speaking of information flow here.

**Reviewer #3 (Public review):**

Summary:

This study investigates the computational role of top-down feedback in artificial neural networks (ANNs), a feature that is prevalent in the brain but largely absent in standard ANN architectures. The authors construct hierarchical recurrent ANN models that incorporate key properties of top-down feedback in the neocortex. Using these models in an audiovisual integration task, they find that hierarchical structures introduce a mild visual bias, akin to that observed in human perception, not always compromising task performance.

Strengths:

The study investigates a relevant and current topic of considering top-down feedback in deep neural networks. In designing their brain-like model, they use neurophysiological data, such as externopyramidisation and hierarchical connectivity. Their brain-like model exhibits a visual bias that qualitatively matches human perception.

We thank the reviewer for their summary and evaluation of our paper’s strengths.

Weaknesses:

While the model is brain-inspired, it has limited bioplausibility. The model assumes a simplified and fixed hierarchy. In the brain with additional neuromodulation, the hierarchy could be more flexible and more task-dependent.

We agree, there are still many facets of top-down feedback that we have not captured here, and the modulation of hierarchy is an interesting example. We have added some consideration of this point to the limitations section of the discussion.

While the brain-like model showed an advantage in ignoring distracting auditory inputs, it struggled when visual information had to be ignored. This suggests that its rigid bias toward visual processing could make it less adaptive in tasks requiring flexible multimodal integration. It hence does not necessarily constitute an improvement over existing ANNs. It is unclear, whether this aspect of the model also matches human data. In general, there is no direct comparison to human data. The study does not evaluate whether the top-down feedback architecture scales well to more complex problems or larger datasets. The model is not well enough specified in the methods and some definitions are missing.

We agree with the reviewer that we have not demonstrated anything like superior performance (since the brain-like network is quite rigid, as noted) nor have we shown better match to human data with the brain-like network. This was not our intended claim. Rather, we demonstrated here simply that top-down feedback impacts behavior of the networks in response to ambiguous stimuli. We have now added statements to the introduction and discussion to make our specific claims (which are supported by our data, we believe) clear.

**Recommendations for the authors:**

**Reviewer #1 (Recommendations for the authors):**

I believe that the work is very nice but not so mature at this stage. Below, you can find some comments that eventually could improve your manuscript.

(1) Intro, last sentence: "Therefore, top-down feedback is a relevant feature that should be considered for deep ANN models in computational neuroscience more broadly." I don't understand what the authors refer to with this sentence. There are numerous models (deep ANNs) that have been used to model the neural activity and are much simpler than the one proposed here which contains very complex models and connectivity. Although I do agree that the top-down connections are very important there is no data to support their importance for modeling the brain.

Respectfully, we disagree with the reviewer that we don’t provide data to demonstrate the importance of top-down feedback for modelling. Indeed, we provided a great deal of data to show that top-down feedback in the networks has real functional implications for behaviour, e.g., it can induce a human-like visual bias. Thus, top-down feedback is a factor that one should care about when modelling the brain. But, we agree with the reviewer that more demonstration of the utility of using top-down feedback for achieving better fits to neural data would be an important next step.

(2) I suggest adding some extra supplementary simulations where, for example, the number of data for visual and auditory pathways is equal in size (i.e., the same number of examples), the number of layers is identical (3 per pathway), and also the number of parameters. Doing this would help strengthen the claims presented in the paper.

In fact, all of the hyperparameters the reviewer mentions here were identical for the different networks, so the experiments the reviewer is requesting here were already part of the paper. We now clarify this in the text.

(3) Results: I suggest adding Tables with quantifications of the presented results. For example, best performance, epochs to converge, etc. As it is now, it is very hard to follow the evidence shown in Figures.

This is a good suggestion, we have now added this table to the start of the supplemental figures.

(4) Figure 2e, 3e: Although VS3, and AS3 have been used only for testing, the plot shows alignments with respect to training epochs. The authors should clarify in the Methods if they tested the network with all intermediate weights during VS1/VS2 or AS1/AS2 training.

Testing scenarios in this context meant that the model was never shown the scenario/task during training, but the models were indeed evaluated on the VS3 and AS3 after each training epoch. We have added clarifications to the figure legends.

(5) Methods: It would be beneficial to discuss how specific hyperparameters were selected based on prior research, empirical testing, or theoretical considerations. Also, it is not clear how the alignment (visual or audio) is calculated. Do the authors use the examples that have been classified correctly for both stimuli or do they exclude those from the analysis (maybe I have missed it).

As noted above, because superior performance was not the goal of this study, we conducted limited hyperparameter tuning. But we have extended the results with additional hyperparameter tuning in a supplementary figure, and describe the hyperparameter choices more thoroughly in the methods. As well, all data includes all model responses, regardless of whether they were correct or not. We now clarify this in the methods.

(6) Code: The code repository lacks straightforward examples demonstrating how to utilize the modeling approach. Given that it is referred to as a "framework", one would expect it to facilitate easy integration into various models and tasks. Including detailed instructions or clear examples would significantly improve usability and help users effectively apply the proposed methodology.

We agree with the reviewer, this would be beneficial. We have revised the README of the codebase to explain the model and its usage more clearly and included an interactive jupyter notebook with example training on MNIST.

Some minor comments are given below. Generally speaking, the Figures need to be more carefully checked for consistent labels, colors, etc.

(1) Page 4, 1st paragraph - grammar correction: "a larger infragranular layer" or "larger infragranular layers"

Thank you for catching this, we have fixed the text.

(2) Page 4, 2nd para - rephrase: "In three additional control ANNs" → "In the third additional control ANN"

In fact, we did mean three additional control ANNs, each one representing a different randomized connectivity profile. We now clarify this in the text and provide the connectivity of the two other random graphs in the supplemental figures.

(3) Page 4, VAE acronym needs to be defined before its first use

The variational autoencoder is introduced by its full name in the text now.

(4) Page 4: Fig. 2c reference should be Fig. 2b, Fig. 2d should be Fig. 2c, Fig. 2b should be Fig. 2d, VS4; Fig. 2b, bottom should be VS4; Fig. 2f, Fig. 2f to Fig. 2g. Double check the Figure references in the text. Here is very confusing for the reader.

We have now fixed this, thank you for catching it.

(5) Page 5, 1st para: "Altogether, our results demonstrated both" → "Altogether, our results demonstrated that both"

This has been updated.

(6) Figure 2: In the e and g panels the x label is missing.

This was actually because the x-axis were the same across the panels, but we see how this was unclear, so we have updated the figure.

(7) Figure 3: There is no panel g (the title is missing); In panels b, c, e, and g the y label is missing, and in panels e and g the x label is missing. Also, the Feedforward model is shown in panel g but it is introduced later in the text. Please remove it from Figure 3. Also in legend: "AV Reverse graph" → "Reverse graph". Also, "Accuracy" and "Alignment" should be presented as percentages (as in Figure 2).

This has been corrected.

(8) Figure 4; x labels are missing.

As with point (6), this was actually because the x-axis were the same across the panels, but we see how this was unclear, so we have updated the figure.

(9) Page 7; I can’t find the cited Figure S1.

Apologies, we have added the supplemental figure (now as S4). It shows the results of models with multiplicative feedback on the task in Fig 5 (as opposed to models with composite feedback shown in the main figure).

**Reviewer #2 (Recommendations for the authors):**

(1) Discussion Section 3.1 is only a literature review, and does not really add any value.

Respectfully, we think it is important to relate our work to other computational work on the role of top-down feedback, and to make clear what our specific contribution is. But, we have updated the text to try to place additional emphasis on our study’s contribution, so that this section is more than just a literature review.

“Our study adds to this previous work by incorporating modulatory top-down feedback into deep, convolutional, recurrent networks that can be matched to real brain anatomy. Importantly, using this framework we could demonstrate that the specific architecture of top-down feedback in a neural network has important computational implications, endowing networks with different inductive biases.”

(2) Including ipython notebooks and some examples would be great to make it easier to use the code.

We now provide a demo of how to use the code base in a jupyter notebook.

(3) The description of the model is hard to comprehend. Please name and describe all parameters. Also, a figure would be great to understand the different model equations.

We have added definitions of all model terms and parameters.

(4) The terminology is not really clear to me. For example "The results further suggest that different configurations of top-down feedback make otherwise identically connected models functionally distinct from each other and from traditional feedforward only recurrent models." The feedforward and only recurrent seem to contradict each other. Would maybe driving and modulatory be a better term here? I also saw in the code that you differentiate between three types of inputs, modulatory, threshold offset and basal (like feedforward). How about you only classify connections based on these three type? I was also confused about the feedforward only model, because I was unsure whether it is still feedback connections but with "basal" quality, or whether feedback connections between modalities and higher-to-lower level layers were omitted altogether.

We take the reviewer’s point here. To clarify this, we have updated the text to refer to “driving only” rather than “feedforward only”, to make it obvious that what we change in these models is simply whether the connection has any modulatory impact on the activity.

(5) "incorporating it into ANNs can affect their behavior and help determine the solutions that the network can discover." -> Do you mean constrain? Overall, I did not really get this point.

Yes, we mean that it constrains the solutions that the network is likely to discover.

(6) "ignore the auditory inputs when they visual inputs were unambiguous" -> the not they

This has been fixed. Thank you for catching it.

(7) xlabel in Figure 4 is missing.

This has been fixed, thank you for catching it.

**Reviewer #3 (Recommendations for the authors):**

Major:

(1) How alignment is computed is not defined. In addition to a proper definition in the methods section, it would be nice to briefly define it when it first appears in the results section.

We’ve added an explicit definition of how alignment is calculated in the methods and emphasized the calculation when its first explained in the results

(2) A connectivity matrix for the feedforward-only model is missing and could be added.

We have added this to Figure 1.

(3) The connectivity matrix for each random model should also be shown.

We’ve shown each of the random model configurations in the new supplemental figure S1.

(4) Initial parameters are not defined, such as W, b etc. A table with all model parameters would be great.

We have added a table to the methods listing all of the parameters.

(5) Would be nice to show the t-sne plots (not just the NH score) for each model and each task in the appendix.

We can provide these figures on request. They massively increase the file size of the paper pdf, as there’s 49 of them for each task and each model, 980 in total. An example t-SNE plot is provided in figure 6.

Minor:

(1) Page 4:

"we refer to this as Visual-dominant Stimulus case 1, or VS1; Fig. 1a, top." This should be Fig. 2a.

(2) "In stimulus condition VS1, all of the models were able to learn to use the auditory clues to disambiguate the images (Fig. 2c)."

This should be Fig. 2b.

(3) "In comparison, in VS2, we found that the brainlike model learned to ignore distracting audio inputs quickly and consistently compared to the random models, and a bit more rapidly than the auditory information (Fig 2d)."

This should be Fig. 2c.

(4) "VS3; Fig. 2b, top"

This should be Fig. 2d

(5) "while all other models had to learn to do so further along in training (Fig. 2e)."

It is not stated explicitly, but this suggests that the image-aligned target was considered correct, and that weight updates were happening.

(6) "VS4; Fig. 2b, bottom"

This should be Fig. 2f

(7) "adept at learning (Fig. 2f)."

This should be Fig. 2g

(8) Figure 3:b,c,e y-labels are missing

3f: both x and y labels are missing

(9) Figure labeling in the text is not consistent (Fig. 1A versus Fig. 2a)

(10) Doubled "the" in ""This shows that the inductive bias towards vision in the brainlike model depended on the presence of the multiplicative component of the the feedback"

(11) Page 9 Figure 6: The caption says b shows the latent spaces for the VS2 task, whereas the main text refers to 6b as showing the latent space for the AS2 task. Please correct which task it is.

(12) Methods 4.1 page 13

"which is derived from the feedback input (h_{l−1})"

This should be h_{l+1}

(13) r_l, u_l, u and c are not defined to which aspects of the model they refer to

Even though this is based on a previous model, the methods section should completely describe the model.

Equations 1,2,3: the notation [x;y] is unclear and should be defined.

Equation 5: u should probably be u_l.

(14) Page 14 typo: externopyrmidisation.

(15) It is confusing to use different names for the same thing: the all-feedforward model, the all feedforward network, the feedforward network, and the feedforward-only model are probably all the same? Consistent naming would help here.

Thank you for the detailed comments! We’ve fixed the minor errors and renamed the feedforward models to drive-only models.
